# A Highly Protective Live-Attenuated Vaccine Generated by Targeted Deletion of the *Mycobacterium bovis* Virulence Factor VapC40

**DOI:** 10.3390/ijms27094067

**Published:** 2026-05-01

**Authors:** Xin Ge, Haoran Wang, Dingpu Liu, Yuhui Dong, Lin Li, Puxiu Shen, Yue Li, Jiaming Zhang, Xiangmei Zhou, Ruichao Yue

**Affiliations:** State Key Laboratory of Veterinary Public Health and Safety, College of Veterinary Medicine, China Agricultural University, Beijing 100193, China

**Keywords:** *Mycobacterium bovis*, toxin-antitoxin system, VapBC40, virulence factor, live-attenuated vaccine

## Abstract

Type II toxin–antitoxin (TA) systems are significantly expanded in the *Mycobacterium tuberculosis* complex; however, the functional role of the VapBC40 system in *Mycobacterium bovis* (*M. bovis*) pathogenesis remains poorly characterized. This study aimed to investigate the role of VapBC40 in mycobacterial virulence and evaluate its potential as a target for rational vaccine attenuation. We performed evolutionary analysis and yeast two-hybrid assays to characterize VapBC40 system specificity, conducted in vitro macrophage infection models and in vivo murine studies to assess virulence contribution, and evaluated the immunoprotective efficacy of a VapC40 knockout strain. Evolutionary analysis revealed progressive sequence conservation and stringent homologous pairing specificity within the VapBC40 system. The VapC40 toxin correlates with enhanced intracellular bacterial survival, increased host cell death, and more severe pulmonary pathology with systemic dissemination. Based on these findings, we evaluated the vaccine potential of a *vapC40* knockout strain. Immunization with this attenuated strain elicited a Th1 cellular immune response, characterized by enhanced IFN-γ production and increased frequency of CD4^+^IFN-γ^+^ T cells. Upon challenge with virulent *M. bovis*, the knockout strain conferred superior protection compared to the conventional BCG vaccine, significantly reducing lung pathology and restricting extrapulmonary bacterial dissemination. Although the molecular mechanisms underlying VapC40-mediated effects remain to be fully elucidated, our findings suggest an important role of the VapBC40 system in mycobacterial-host interactions and support its potential as a target for next-generation tuberculosis vaccine development.

## 1. Introduction

Tuberculosis (TB), caused by members of the *Mycobacterium tuberculosis* complex (MTBC), including *Mycobacterium tuberculosis* (*M. tuberculosis*) and *Mycobacterium bovis* (*M. bovis*), remains one of the leading infectious disease killers worldwide, affecting millions of individuals annually [1]. Tuberculosis remains a major global health challenge, with an estimated 10.7 million new cases worldwide in 2024 (incidence rate: 131 per 100,000 population). The disease burden is highly concentrated geographically, with just eight countries accounting for two-thirds (67%) of all global cases, led by India (25%) and Indonesia (10%) [1]. A characteristic feature of MTBC pathogens is their remarkable capacity to survive, adapt, and replicate within host macrophages, thereby establishing persistent infections and facilitating disease progression [2,3]. Currently, the Bacillus Calmette–Guérin (BCG) vaccine represents the only licensed vaccine available for TB prevention worldwide [4,5]. However, its protective efficacy against pulmonary TB in adults is highly variable and geographically dependent, with effectiveness ranging from 20 to 50% in low-income regions compared to nearly 100% in high-income countries [4,6]. This variation is particularly evident in equatorial regions of Africa and South Asia, where the TB burden is highest [7,8]. The vaccine provides suboptimal protection in vulnerable populations, particularly adults, HIV-infected individuals who face an 18-fold increased risk of active TB, and other immunocompromised patients [7,9]. Furthermore, BCG offers limited protection against latent TB infection progression and extrapulmonary disease manifestations, with significant protective effects observed primarily in children under 5 years of age [10]. These inherent limitations underscore the urgent need to identify critical virulence determinants of MTBC pathogens and to develop rationally designed, live-attenuated vaccines capable of inducing more robust and durable protective immunity compared to the current BCG vaccine.

Type II toxin–antitoxin (TA) systems are significantly amplified in the MTBC genome and play crucial roles in stress adaptation and persistence [11,12]. A canonical type II TA module consists of a stable toxin protein and a labile antitoxin that neutralizes toxin activity under normal growth conditions. Upon environmental stress, including oxidative/nitrosative stress, chemical stress, and nutrient starvation, proteolytic degradation of the antitoxin liberates the toxin, which then interferes with cellular processes such as transcription, translation, and cell division through ribonuclease activity, resulting in growth arrest and metabolic reprogramming that promotes bacterial survival and establishment of persistence under harsh conditions [13,14,15]. The VapBC family constitutes the most abundant class of TA systems in MTBC, with over 50 members identified [12]. Recent studies have revealed diverse functions of specific VapBC systems in mycobacterial biology. For instance, VapBC22 has been implicated in regulating *M. tuberculosis* pathogenicity and modulating host immune responses [16]. VapBC13 and VapBC26 mediate bacterial growth inhibition and oxidative stress responses through specific tRNA cleavage mechanisms [17]. Additionally, *M. abscessus* VapC5 promotes antibiotic resistance by inducing ribosome overexpression and activating multidrug resistance pathways, thereby facilitating bacterial escape from antibiotic killing [18]. Despite these advances in understanding individual VapBC family members, the specific contribution of VapBC40 to *M. bovis* pathogenesis and its potential role as a virulence determinant remain poorly defined.

In this study, we aimed to comprehensively examine the role of the VapBC40 system in *M. bovis* pathogenesis and evaluate its potential as a target for rational vaccine development. Our objectives were to: (1) investigate the evolutionary conservation and protein–protein interaction specificity of VapBC40 through phylogenetic analysis and interaction assays; (2) assess the contribution of VapC40 to bacterial virulence and host pathology using both in vitro and in vivo experimental approaches; and (3) evaluate the immunogenicity and protective efficacy of a rationally designed VapC40 knockout strain as a potential vaccine candidate. Through these investigations, we sought to provide novel insights into the role of VapBC40 in mycobacterial pathogenesis and determine its potential as a promising target for rational vaccine attenuation and next-generation tuberculosis vaccine development.

## 2. Results

### 2.1. Evolutionary Conservation and Functional Specificity of the Vapbc40 Toxin-Antitoxin System in Mycobacteria

To investigate the evolutionary dynamics of the VapBC40 system within the Mycobacterium genus and its association with pathogenic adaptation, we conducted multiple sequence alignment across four representative mycobacterial species with different evolutionary backgrounds and pathogenic characteristics. We selected *M. tuberculosis* and *M. bovis* as highly specialized obligate intracellular pathogens, *M. lacus* as a phylogenetically related non-tuberculous mycobacterium (NTM) representing an evolutionary intermediate, and *Mycolicibacterium doricum* as a distantly related environmental NTM. Through comparative analysis of VapC40 and VapB40 protein sequence conservation and variation, we sought to elucidate the molecular evolutionary patterns of this system during the transition from environmental saprophytes to intracellular pathogens.

**Figure 1:**
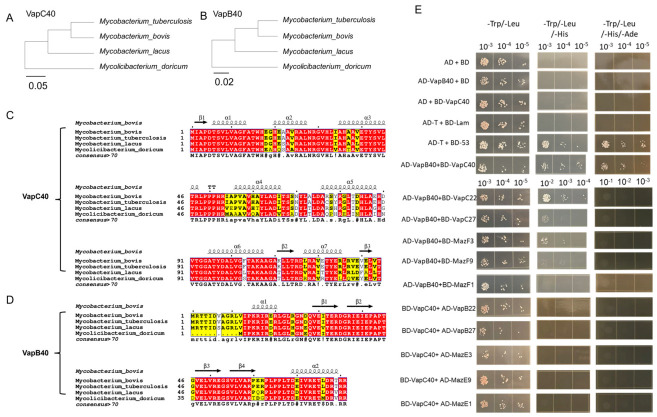
Sequence conservation and strict homologous pairing specificity of the VapBC40 system. (**A**,**B**) Phylogenetic trees of VapC40 (**A**) and VapB40 (**B**) proteins across representative mycobacterial species. (**C**,**D**) Multiple sequence alignment visualization of VapC40 (**C**) and VapB40 (**D**) proteins from representative mycobacterial species. The alignments were visualized using ESPript 3.0. Residues showing 100% identity are highlighted in red backgrounds, while highly similar residues are indicated in yellow. Dot above the sequences serve as position markers, placed at every 10th residue (i.e., positions 1, 10, 20, 30, …) to facilitate residue numbering; they carry no biological significance and are used solely for positional reference. (**E**) Specificity of the VapB40–VapC40 interaction assessed by a yeast two-hybrid (Y2H) assay. Yeast strains co-transformed with the indicated toxin and antitoxin combinations were spotted onto auxotrophic selective media. Growth indicates a positive protein–protein interaction.

Structural visualization and sequence alignment revealed distinct conservation patterns (Figure 1A–D). Using *M. doricum* as the evolutionary reference point, the sequence identities of VapC40 and VapB40 proteins with their *M. bovis* counterparts were 77.78% and 75.31%, respectively, with notable sequence deletions observed. This suggests relaxed selective constraints during environmental adaptation, permitting structural diversity and varied autoregulatory mechanisms. In *M. lacus*, these sequence deletions were absent, and the sequence identities increased to 82.84% for VapC40 and 90.12% for VapB40. This pattern indicates intensified selective pressure on core amino acid sequences as bacteria adapted to host-associated niches. Within the MTBC, VapB40 sequences showed complete conservation (100% identity), while VapC40 exhibited near-perfect conservation (99.25% identity), differing by a single amino acid substitution. These observations reveal the evolutionary trajectory of the VapBC40 system and its correlation with pathogenic specialization. The high degree of sequence conservation within MTBC suggests substantial selective pressure during host adaptation.

To examine the pairing specificity between VapB40 and VapC40 proteins, we employed yeast two-hybrid cross-validation assays. Based on phylogenetic relationships among toxin-antitoxin family members, we selected closely related type II TA system components for heterologous interaction testing [19,20]. The antitoxin VapB40 was tested against heterologous toxins including VapC22, VapC27, MazF3, MazF9, and MazF1, while toxin VapC40 was examined with heterologous antitoxins including VapB22, VapB27, MazE3, MazE9, and MazE1. Growth analysis on selective media demonstrated that among all tested combinations, only the cognate VapB40–VapC40 pair supported robust clonal growth (Figure 1E). No detectable interactions were observed in heterologous pairings. These findings demonstrate the high specificity of VapB40–VapC40 protein interactions and their strict homologous pairing requirement.

### 2.2. Vapc40 Enhances Mycobacterium Bovis Intracellular Survival and Induces Macrophage Cell Death

**Figure 2:**
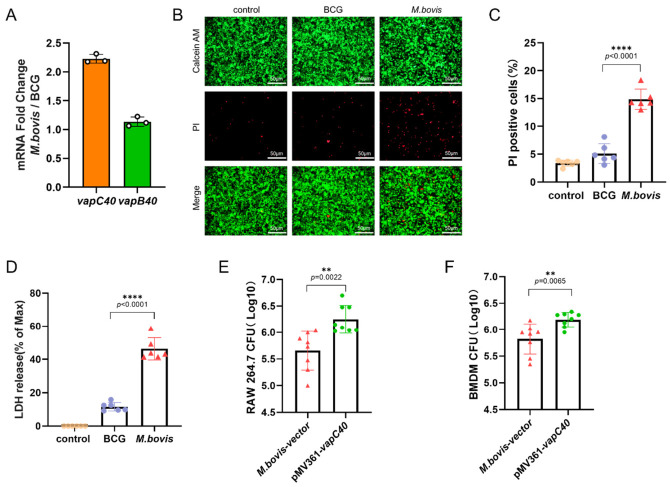
VapC40 promotes mycobacterial intracellular survival. (**A**) Relative transcriptional levels of the *vapBC40* genes in *M. bovis* and BCG. (**B**) Representative fluorescence micrographs of RAW 264.7 cells stained with Calcein AM/PI at 24 h post-infection with *M. bovis* or BCG. Live cells display green fluorescence (Calcein AM), while dead cells exhibit red fluorescence (PI). Scale bar = 50 μm. (**C**) Statistical quantification of the percentage of dead cells based on the fluorescence images in (**B**). (**D**) Cytotoxicity induced by *M. bovis* and BCG in RAW 264.7 cells at 24 h post-infection, as determined by the LDH release assay. (**E**,**F**) Intracellular bacterial survival assessed by colony-forming unit (CFU) enumeration in RAW 264.7 cells (**E**) and primary BMDMs (**F**) infected with the *M. bovis* empty vector control or the *vapC40*-overexpressing strain for 24 h. (Data are presented as mean ± SD; statistical significance was determined by *t*-test. Asterisks indicate statistical significance: ** *p* < 0.01, **** *p* < 0.0001; non-significant differences *p* ≥ 0.05 are not labeled).

To determine whether the evolutionary adaptation of the TA system is reflected in virulent versus attenuated strains, we observed that the transcriptional level of *vapC40* in the naturally virulent *M. bovis* was significantly higher than that in the artificial attenuated vaccine strain BCG (Figure 2A). While our current data cannot definitively distinguish whether this differential expression stems from genetic background differences or regulatory variations, the elevated vapC40 transcription in the virulent strain implies a close relationship between VapC40 and host adaptation mechanisms. To verify whether this elevated transcription translates into a cellular virulence advantage, we evaluated the direct impact of *M. bovis* and BCG on macrophage viability using an in vitro infection model. Compared to the BCG group, *M. bovis* infection induced more severe host cytopathy. Specifically, the proportion of cell death was significantly higher in the *M. bovis* group (Figure 2B,C), accompanied by a substantial increase in LDH release in the culture supernatant (Figure 2D), indicating profound cell membrane permeabilization and necrosis. In contrast, BCG infection elicited relatively low levels of cell death and LDH release. Combined with the specific upregulation of *vapC40* in *M. bovis*, these phenotypic differences preliminarily suggest that the high-level expression of VapC40 likely mediates *M. bovis* virulence within macrophages, acting as a key pathogenic factor driving host cell damage.

Having observed that virulent *M. bovis* induces stronger host cell damage, we further elucidated the specific role of VapC40 during macrophage infection by constructing a VapC40-overexpressing recombinant *M. bovis* strain. Both the RAW 264.7 and BMDMs, which more closely resemble the physiological in situ state, were employed as in vitro infection models. Host cells were infected with either the VapC40-overexpressing strain or the control strain at an identical multiplicity of infection (MOI = 20). At 24 h post-infection, the intracellular survival of the bacteria was quantified by assessing CFUs via agar plating. The results demonstrated that VapC40 overexpression significantly enhanced the intracellular survival capacity of *M. bovis* in both passaged RAW 264.7 cells (Figure 2E) and primary BMDM models (Figure 2F). Compared with the control group, the VapC40-overexpressing strain exhibited a significant increase in CFU counts within both macrophage types. Coupled with its cytopathic phenotype, these findings strongly indicate that VapC40 effectively assists the pathogen in evading immune clearance and serves as a critical effector driving *M. bovis* intracellular virulence.

### 2.3. Overexpression of Vapc40 Exacerbates Mycobacterium Bovis Pathogenesis In Vivo

**Figure 3:**
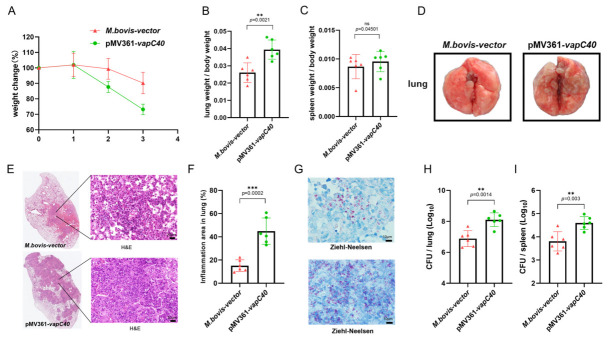
VapC40 exacerbates pulmonary pathology and promotes systemic dissemination in a murine infection model. (**A**) Body weight changes of C57BL/6 mice monitored weekly following intranasal (i.n.) infection with the *M. bovis* empty vector control or the *vapC40*-overexpressing strain. (**B**,**C**) Organ indices for the lungs (**B**) and spleens (**C**) at 28 days post-infection (dpi), calculated as the ratio of the respective organ weight to the total body weight. (**D**) Representative images of gross pulmonary pathology at 28 dpi. (**E**) Histopathological evaluation of lung sections stained with hematoxylin and eosin (H&E). Scale bar, 20 μm. (**F**) Quantification of the inflamed area, expressed as the ratio of the inflammatory lesion area to the total lung section area based on the H&E-stained images. (**G**) Representative Ziehl-Neelsen (ZN) acid-fast staining of lung sections. Acid-fast bacilli (*M. bovis*) are visibly stained pink. Scale bar, 10 μm. (**H**,**I**) Bacterial burdens evaluated by viable colony-forming unit (CFU) enumeration in the lungs (**H**) and spleens (**I**) of the infected mice at 28 dpi. (Data are presented as mean ± SD; statistical significance was determined by *t*-test. Asterisks indicate statistical significance: ** *p* < 0.01, *** *p* < 0.001; ns, not significant *p* ≥ 0.05).

To comprehensively evaluate the impact of VapC40 on the virulence of *M. bovis* in vivo, we established a mouse infection model for systematic verification. Four weeks post-infection, mice infected with the VapC40-overexpressing strain exhibited significant progressive weight loss compared to those inoculated with the empty vector control strain (Figure 3A). Furthermore, the lung-to-body weight ratio was significantly elevated in the overexpression group (Figure 3B), whereas the spleen-to-body weight ratio showed no significant difference (Figure 3C). Gross pathological observations revealed that the overexpressing strain exacerbated *M. bovis*-induced pulmonary lesions; compared to the control group, the lungs of mice infected with the overexpressing strain displayed large, coalescing nodules and an irregular, uneven surface (Figure 3D).

Histopathological examination of lung tissues (H&E staining) demonstrated severe structural destruction in the lungs of mice infected with the VapC40-overexpressing strain. The parenchyma showed dense infiltration of massive inflammatory cells and typical patchy necrotic areas (Figure 3E). In contrast, pulmonary lesions in the empty vector control group were relatively mild, with more localized inflammatory infiltration. To objectively quantify the pathological damage induced by VapC40, whole-slide digital scanning was performed on the lung sections of each group, and the inflammatory area across the entire section was quantified using image analysis software. The results indicated a significant increase in the percentage of inflammatory lesion area in the lungs of the VapC40-overexpressing group compared to the control group (Figure 3F). These histopathological phenotypes strongly suggest that the high-level expression of VapC40 drastically exacerbates the inflammatory response and parenchymal organ damage induced by *M. bovis* in the host lungs.

To observe the in situ distribution of the pathogen within the lesioned tissues, lung sections were further subjected to acid-fast staining. Microscopic examination revealed that in the VapC40-overexpressing group, acid-fast positive *M. bovis* bacilli were widely scattered in massive absolute numbers (Figure 3G). Conversely, the distribution of mycobacteria in the control group was more restricted, with a visibly lower local bacterial load. To precisely quantify viable bacterial colonization and systemic dissemination, we concurrently determined colony-forming units (CFUs) in the primary target organ, the lungs, and the peripheral immune organ, the spleen. Statistical analysis revealed that the viable bacterial load in the lungs of mice infected with the VapC40-overexpressing strain was significantly higher than that of the control group (Figure 3H). More importantly, the CFU counts in the spleen also exhibited a substantial increase (Figure 3I). This dramatic elevation in splenic bacterial burden not only corroborates the morphological observations from acid-fast staining but also directly proves that VapC40 confers to *M. bovis* a stronger proliferative advantage at the primary infectious foci and an enhanced pathogenic capacity to breach tissue barriers for extrapulmonary dissemination.

### 2.4. Immunization with the Vapc40 Knockout Strain Elicits Robust Th1-Biased Cellular Immune Responses

**Figure 4:**
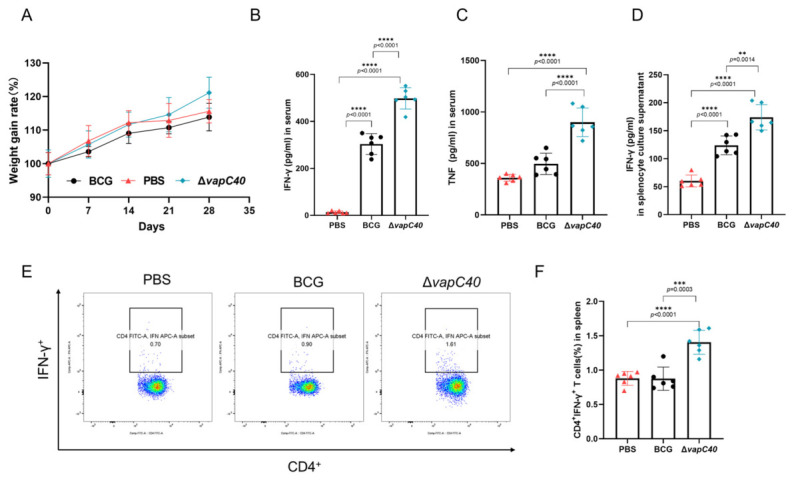
Evaluation of the immunogenicity of the live-attenuated *vapC40* knockout strain in mice. (**A**) Body weight changes of mice monitored weekly throughout the 28-day immunization period. (**B**,**C**) Serum cytokine levels of IFN-γ (**B**) and TNF-α (**C**) at 28 days post-immunization, as determined by ELISA. (**D**) IFN-γ secretion levels in culture supernatants of antigen-stimulated splenocytes at 28 days post-immunization, as quantified by ELISA. (**E**,**F**) Representative flow cytometry pseudocolor plots (**E**) and statistical quantification (**F**) illustrating the frequencies of antigen-specific CD4^+^IFN-γ^+^ T cells in the spleens of the immunized mice. (Data are presented as mean ± SD; Statistical significance was determined by one-way ANOVA followed by Tukey’s multiple comparison test. Asterisks indicate statistical significance: ** *p* < 0.01, *** *p* < 0.001, **** *p* < 0.0001; non-significant differences *p* ≥ 0.05 are not labeled).

Based on our previous findings that deletion of the *vapC40* gene significantly attenuates in vivo virulence and dissemination in *M. bovis*, we hypothesized that this knockout strain could serve as a novel live-attenuated vaccine. To systematically evaluate its immunogenicity, we subcutaneously immunized mice using the traditional BCG vaccine as a standard control. During the immunization period, continuous monitoring of mouse body weight revealed normal growth in the knockout strain-immunized group, preliminarily confirming the safety of this strain (Figure 4A). Four weeks post-immunization, peripheral blood was collected to isolate serum, and the levels of key effector cytokines in the circulation were quantified using ELISA. The results demonstrated that the serum IFN-γ and TNF-α concentrations in the knockout strain-immunized mice were significantly higher than those in the BCG control group (Figure 4B,C). As a core cytokine essential for activating macrophage bactericidal functions, the substantial elevation of serum IFN-γ and TNF-α preliminarily suggests that the knockout strain elicited a more robust systemic immune response in vivo.

Protective immunity against tuberculosis relies heavily on antigen-specific T cell responses. Therefore, we further aseptically isolated the spleens of the immunized mice, prepared single-cell suspensions, and subjected them to in vitro restimulation with antigens. Flow cytometry analysis revealed that the proportion of CD4^+^IFN-γ^+^ T lymphocytes in the splenocytes of the knockout strain-immunized group was significantly higher than that of the BCG group (Figure 4E,F). Concurrently, analysis of the culture supernatants from the antigen-stimulated splenocytes confirmed an extremely significant upregulation of IFN-γ secretion in the knockout group (Figure 4D). Collectively, these findings explicitly indicate that the knockout strain successfully and efficiently activated a more potent immune response within the host, specifically inducing a protective anti-tuberculosis immunity dominated by Th1-type cellular responses.

### 2.5. The Vapc40 Knockout Strain Provides Protective Efficacy Against M. bovis Challenge

**Figure 5:**
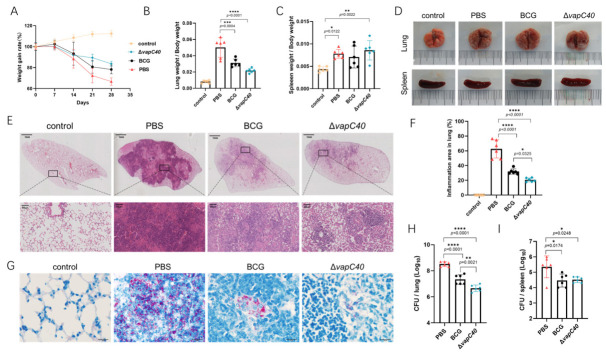
The *vapC40*-deficient strain confers robust protection against *M. bovis* infection in vivo. (**A**) Body weight changes of the immunized mice monitored weekly following the virulent *M. bovis* challenge. (**B**,**C**) Organ indices of the lungs (**B**) and spleens (**C**) evaluated at 28 days post-challenge, calculated as the ratio of organ weight to total body weight. (**D**) Representative images of gross pulmonary pathology from the challenged mice. (**E**) Histopathological evaluation of the lung sections stained with hematoxylin and eosin. Scale bar, 50 μm (**H**,**E**). (**F**) Statistical quantification of the inflamed area, expressed as the ratio of the inflammatory lesion area to the total lung section area based on the H&E staining. (**G**) Representative Ziehl-Neelsen (ZN) acid-fast staining of the lung sections. The *M. bovis* bacilli are visibly stained pink. Scale bar, 10 μm. (**H**,**I**) Viable bacterial burdens evaluated by colony-forming unit (CFU) enumeration in the lungs (**H**) and spleens (**I**) of the mice at 28 days post-challenge. (Data are presented as mean ± SD; Statistical significance was determined by one-way ANOVA followed by Tukey’s multiple comparison test. Asterisks indicate statistical significance: * *p* < 0.05, ** *p* < 0.01, *** *p* < 0.001, **** *p* < 0.0001; non-significant differences *p* ≥ 0.05 are not labeled).

To comprehensively evaluate the actual protective efficacy of the knockout strain as a vaccine candidate, an in vivo mouse challenge experiment was conducted. Four weeks after subcutaneous immunization, mice in each group were challenged with virulent *M. bovis* via the intranasal route. Systematic pathological and bacteriological evaluations were performed four weeks post-infection. Continuous physiological monitoring during the challenge period revealed that, compared to the traditional BCG-immunized control group, immunization with the knockout strain significantly alleviated the progressive weight loss induced by *M. bovis* infection (Figure 5A). Concurrently, macroscopic evaluations revealed that the lung organ index of this group was maintained at a significantly lower level, indicating that the immunization effectively mitigated severe infection-induced pulmonary swelling (Figure 5B), whereas the spleen organ index exhibited no significant difference (Figure 5C).

Gross pathological observations of the organs visually corroborated the improvement in the aforementioned physiological indicators: compared to the BCG group, the number of macroscopic tubercular nodules on the lung surfaces of the knockout strain-immunized mice was drastically reduced, and the tendency for lesion coalescence was significantly attenuated (Figure 5D). Microscopic histopathological analysis clearly showed that the area of inflammatory cell infiltration in the lung parenchyma of the knockout strain-immunized group was markedly reduced, and the typical tissue necrosis was significantly milder than that in the BCG group (Figure 5E,F). This indicates that the highly efficient immune response elicited by the knockout strain successfully contained the severe local inflammation and substantial tissue destruction triggered by the virulent strain.

In agreement with the histopathological evaluation, Ziehl-Neelsen acid-fast staining of the lung sections revealed a visibly reduced accumulation of mycobacteria in the knockout strain-immunized group (Figure 5G). To further establish a direct quantitative relationship between immune protection and pathogen clearance capacity, the viable CFU in the target organs of the mice was precisely determined. The results conclusively demonstrated that the pulmonary bacterial load in the mice immunized with the knockout strain was significantly lower than that of the BCG group (Figure 5H). Furthermore, bacterial dissemination to the spleen was also profoundly restricted (Figure 5I). These critical bacteriological data fully illustrate that the immune memory defense line mediated by the knockout strain not only effectively restricted the local proliferation of the virulent strain at the portal of entry but also potently impeded the systemic dissemination of the pathogen across anatomical barriers to peripheral organs.

## 3. Discussion

In the present study, we provide evidence that the conserved VapBC40 toxin-antitoxin system contributes to *M. bovis* pathogenesis and represents a potential target for vaccine attenuation. Through systematic investigation of its evolutionary dynamics, strict homologous pairing, and in vivo phenotypic effects, we demonstrated that the VapC40 toxin enhances intracellular bacterial survival and promotes systemic dissemination. These findings are consistent with previous studies showing that toxin-antitoxin systems facilitate bacterial persistence under stress conditions [15,16,17]. Building upon these mechanistic insights, we evaluated the therapeutic potential of targeted *vapC40* gene deletion. The resulting attenuated strain exhibited enhanced immunogenicity, eliciting Th1 cellular immunity characterized by increased IFN-γ production, which is crucial for anti-mycobacterial immunity [21]. In murine challenge studies, this knockout strain demonstrated improved protective efficacy compared to the standard BCG vaccine, reducing pulmonary pathology and limiting extrapulmonary bacterial spread. While the precise molecular mechanisms underlying VapC40-mediated virulence require further elucidation, our findings contribute to understanding the role of TA systems in mycobacterial pathogenesis and suggest the potential utility of rationally designed attenuated strains for tuberculosis vaccine development.

The evolutionary transition of mycobacteria from environmental saprophytes to obligate intracellular pathogens is typically accompanied by substantial genomic reduction and selective retention of essential survival determinants [22,23,24]. Our phylogenetic analysis revealed progressive sequence conservation of the VapBC40 system toward the MTBC. Unlike the structurally variable sequences observed in environmental NTM, the high degree of conservation of VapBC40 within MTBC suggests that this module experienced substantial positive selective pressure during host adaptation. The considerable expansion of type II TA systems in the MTBC genome raises questions regarding potential functional redundancy and regulatory interactions [11]. However, our yeast two-hybrid assays demonstrated strict homologous pairing specificity between VapB40 and VapC40, with no detectable cross-interactions with other closely related toxin-antitoxin members. This exclusive interaction pattern is biologically significant, as it ensures precise, independent activation of the VapC40 toxin in response to specific intramacrophage stresses while preventing undesired signaling interference within the complex mycobacterial TA network. The combination of sequence conservation and functional specificity may provide the evolutionary basis for VapC40 to function as a specialized virulence factor in host–pathogen interactions.

Building upon this evolutionary and functional specialization, our in vitro and in vivo models provided evidence for VapC40 as a contributor to mycobacterial virulence. During intracellular infection, VapC40 overexpression enhanced *M. bovis* survival within both continuous and primary macrophage models, accompanied by increased host cell death. In the context of mycobacterial infection, the induction of macrophage necrosis represents an immune evasion strategy that allows pathogens to escape the hostile intramacrophage antimicrobial environment and facilitates cell-to-cell spread [25]. These in vitro observations were consistent with our murine infection model findings. Enhanced VapC40 expression exacerbated pulmonary pathological damage, characterized by increased inflammatory infiltration and tissue necrosis. Furthermore, it promoted extrapulmonary dissemination, as demonstrated by elevated splenic bacterial burden compared to control strains. We hypothesize that VapC40-mediated macrophage destruction may compromise the integrity of local tissue barriers in the lungs, potentially facilitating bacterial dissemination to distant sites, though this proposed mechanism requires direct experimental validation. While our findings are consistent with VapC40 functioning as an important virulence factor that influences host–pathogen interaction dynamics, future studies specifically designed to examine macrophage viability, tissue barrier integrity, and bacterial dissemination pathways will be necessary to confirm this hypothetical relationship and its contribution to disease progression.

Based on the established role of VapC40 in mycobacterial virulence, we sought to explore whether this mechanistic insight could inform rational vaccine design. The goal of live-attenuated vaccines is to achieve an appropriate balance between safety through attenuation and effective stimulation of protective immunity [26]. Our results suggest that targeted deletion of the *vapC40* gene may generate a vaccine candidate with potentially enhanced immunological properties compared to the traditional BCG vaccine in our experimental model. Immunologically, the knockout strain elicited enhanced Th1 cellular responses, characterized by increased serum IFN-γ and TNF-α levels and a higher frequency of antigen-specific CD4^+^IFN-γ^+^ T cells. This Th1 polarization is considered essential for effective anti-tuberculosis immunity [27]. Furthermore, this enhanced immunogenicity corresponded with improved protective efficacy during virulent *M. bovis* challenge in our short-term study. Compared to BCG-immunized mice, those vaccinated with the knockout strain exhibited reduced viable bacterial burdens in both lungs and spleen, along with decreased histopathological necrosis. We propose that the absence of VapC40 toxin may reduce the strain’s capacity to rapidly destroy host macrophages and disrupt tissue barriers. This attenuation potentially extends the duration for efficient antigen presentation and dendritic cell activation, allowing development of robust immune responses while minimizing pathogen-induced tissue damage. These characteristics suggest that the *vapC40* knockout strain represents a promising alternative to current BCG vaccination strategies.

Although the precise pathogenic mechanisms of VapC40 remain to be fully elucidated, this study indicates that the highly conserved VapC40 is an important driver of *M. bovis* pathogenesis. We hypothesize that autophagy, apoptosis, or phagosome-related pathways may be involved in the observed macrophage dysfunction. Future mechanistic studies should investigate VapC40’s effects on these cellular processes, including phagosome-lysosome fusion, autophagy regulation, and apoptotic pathway activation. Our preliminary results suggest that rationally deleting this specific virulence module yields a live-attenuated vaccine candidate with potentially improved immunogenicity and protective efficacy compared to the conventional BCG vaccine in our experimental model. Understanding these molecular interactions would provide valuable insights for rational vaccine design and therapeutic target identification. While these initial findings are encouraging, extensive additional validation studies would be necessary before considering clinical development. Collectively, these findings contribute to our understanding of mycobacterial immune evasion and suggest a potential strategy that may warrant further investigation for tuberculosis vaccine improvement.

## 4. Materials and Methods

### 4.1. Ethics Statement

All animal experiments were conducted in accordance with the Guidelines for the Care and Use of Laboratory Animals issued by the Ministry of Science and Technology of China. All experimental protocols were approved by the Institutional Animal Care and Use Committee (IACUC, Approval No. 20110611-01) and the Animal Ethics Committee of China Agricultural University (Approval No. AW02110202–2) on 20 November 2020.

### 4.2. Sequence Alignment and Structural Visualization

The amino acid sequences of VapC40 and VapB40 from representative mycobacterial strains were retrieved from the UniProt database [28]. The three-dimensional structure of *M. bovis* was obtained from the Protein Data Bank (PDB). Multiple sequence alignments were performed using MEGA software (https://www.megasoftware.net/, access on 8 April 2026) [29]. The alignment results, together with the corresponding PDB structure, were visualized using the ESPript 3.0 web server to display sequence conservation in the context of secondary structural elements [30].

### 4.3. Bacterial Strains, Plasmids, and Culture Conditions

*M. bovis* strain C68004 was obtained from the China Institute of Veterinary Drug Control (CVCC, Beijing, China). *M. bovis* strain C68004 has been extensively characterized in our previous studies [31]. Comparative virulence analysis with the clinical isolate N# demonstrated that C68004 produced significant pathological changes at 28 days post-infection with an intranasal dose of 2 × 10^3^ CFU. However, it exhibited lower virulence than the clinical isolate. Based on these virulence properties, the infection dose of 2 × 10^3^ CFU was selected for the current study to ensure reproducible pathogenesis while maintaining animal welfare standards. The vectors used for gene knockout and complementation were kindly provided by Dr. Yicheng Sun (Institute of Pathogen Biology, Chinese Academy of Medical Sciences), and the overexpression constructs were maintained in our laboratory.

Mycobacteria were routinely cultured at 37 °C in Middlebrook 7H9 liquid medium (Difco, BD Biosciences, Sparks, MD, USA) supplemented with 10% (*v*/*v*) oleic acid–albumin–dextrose–catalase (OADC) and 0.05% (*v*/*v*) Tween-80 (Sigma-Aldrich, St. Louis, MO, USA), or on Middlebrook 7H10 agar supplemented with 10% OADC. When required, kanamycin was added at a final concentration of 25 μg/mL for plasmid selection. For both in vitro and in vivo infection experiments, bacteria were harvested during the exponential growth phase (OD600 = 0.3–0.8).

The vapC40 knockout strain was constructed using CRISPR-Cas9-mediated gene deletion following established protocols [32]. Competent *M. bovis*::pNHEJ-recX cells were transformed with the knockout plasmid containing vapC40-specific sgRNA and selected using dual antibiotic resistance. Gene deletion was confirmed by PCR amplification using flanking primers, agarose gel electrophoresis, and DNA sequencing verification. Genetic stability was assessed through serial passage, and transcriptional analysis confirmed the absence of polar effects on neighboring genes.

Yeast two-hybrid assays were performed following established protocols [33]. VapC40 and VapB40 genes were cloned into pGBKT7 (BD fusion) and pGADT7 (AD fusion) vectors, respectively. The resulting constructs were co-transformed into Saccharomyces cerevisiae strain AH109 using the lithium acetate transformation method. To assess interaction specificity, multiple control transformations were performed: (1) empty pGBKT7 and pGADT7 vectors as negative controls, (2) pGADT7 vector co-transformed with BD-VapC40, (3) pGBKT7 vector co-transformed with AD-VapB40, and (4) pGBKT7-53 and pGADT7-T as positive controls. Transformants were first selected on double dropout medium (SD/-Trp/-Leu) to confirm successful co-transformation. Protein–protein interactions were then evaluated by transferring colonies to triple dropout medium (SD/-Trp/-Leu/-His) and quadruple dropout medium (SD/-Trp/-Leu/-His/-Ade), followed by incubation at 30 °C for 3–5 days. While growth on triple dropout medium indicated successful transformation and potential interaction, growth on quadruple dropout medium served as the definitive criterion for positive protein interaction. In contrast, the absence of growth indicated the absence of an interaction.

### 4.4. Quantitative Real-Time PCR

Total RNA from *M. bovis* was isolated using the HiBind Bacterial RNA Kit (Cat# TR2310-02; Magen Biotechnology, Guangzhou, China) according to the manufacturer’s instructions. The purified RNA was reverse-transcribed into complementary DNA (cDNA) using a commercial reverse transcription kit (Cat# R333-01; Vazyme Biotech Co., Ltd., Nanjing, China). Quantitative real-time PCR (RT-qPCR) was performed to assess the transcriptional levels of target genes. The housekeeping gene *sigA* was used as an internal control for normalization [34]. Relative gene expression levels were calculated using the 2^−ΔΔCt^ method.

Primer sequences were as follows:

*sigA*-F: 5′-ACGAAGACCACGAAGACCTCGAA-3′;

*sigA*-R: 5′-GTAGGCGCGAACCGAGTCGGCGG-3′;

*vapC40*-F: 5′-GCGGCTGTGGAAACCTATT-3′;

*vapC40*-R: 5′-GTAGTTGCTGGAGGTGATGTC-3′;

*vapB40*-F: 5′-AACGACCAGGTGGAGATCA-3′;

*vapB40*-R: 5′-CGAACGATTTCGTCGGTCAA-3′.

### 4.5. Cell Culture and In Vitro Infection Model

The murine RAW 264.7 macrophage cell line was obtained from the Cell Culture Center of Peking Union Medical College (Beijing, China) and cultured in Dulbecco’s Modified Eagle Medium (DMEM) supplemented with 10% fetal bovine serum (FBS; Gibco, Grand Island, NY, USA) and 1% penicillin–streptomycin (Sigma-Aldrich, St. Louis, MO, USA) at 37 °C in a humidified atmosphere with 5% CO_2_. BMDM were prepared and cultured as described previously [25]. Primary bone marrow-derived macrophages (BMDMs) were isolated from 6- to 8-week-old specific-pathogen-free (SPF) mice and cultured in RPMI-1640 medium containing 10% FBS and 1% penicillin–streptomycin under the same conditions.

For in vitro infection assays, RAW 264.7 cells and BMDMs were resuspended in their respective media containing 2% FBS and seeded into culture plates [35]. Cells were infected with live *M. bovis* at a defined multiplicity of infection (MOI = 20) in the absence of antibiotics. After incubation at 37 °C for 4 h to allow bacterial internalization, extracellular bacteria were removed by washing the cells three times with pre-warmed sterile phosphate-buffered saline (PBS). The infected macrophages were then maintained in DMEM or RPMI-1640 containing 2% FBS until the indicated time points. At 24 h post-infection, cells were lysed using 0.1% Triton X-100, and the lysates were subjected to 10-fold serial dilutions in PBS. One hundred microliters of each dilution was plated in triplicate on 7H10 agar plates supplemented with OADC. Following incubation at 37 °C for 2–3 weeks, colony-forming units (CFUs) were enumerated from plates containing clearly distinguishable individual colonies. Intracellular bacterial survival was quantified by multiplying colony counts by the appropriate dilution factor and expressing results as log10 CFU per milliliter.

### 4.6. Cell Viability and Cytotoxicity Assays

For the Live/Dead assay, infected macrophages were washed with PBS and stained with a Calcein AM/propidium iodide (PI) working solution (Cat# C2015M; Beyotime, Shanghai, China) at 37 °C for 30 min. Fluorescence images were acquired using a BDS400 inverted fluorescence microscope (Aote Optical, Chongqing, China). The proportion of dead cells was quantified by counting green (live) and red (dead) cells across multiple fields using ImageJ software [31]. In addition, LDH release into the culture supernatants was measured to evaluate cell membrane integrity using an LDH Cytotoxicity Assay Kit (Cat# C0018M; Beyotime, Shanghai, China). The collected supernatants were incubated with the reaction mixture according to the manufacturer’s instructions. After a 30-min incubation in the dark, absorbance at 490 nm was measured using a TECAN Spark multimode microplate reader [31].

### 4.7. Animal Models of Infection and Immunization

Specific-pathogen-free (SPF) female C57BL/6 mice (6–8 weeks old with body weight 18–20 g) were obtained from SPF Biotechnology Co., Ltd. (Beijing, China). A total of 54 mice were acclimatized for one week before being randomly assigned to experimental groups with 6 animals per group. While investigators could not be blinded to treatment groups due to the distinct nature of vaccine strains and experimental design, all subsequent procedures were conducted following standardized protocols to minimize potential bias. To evaluate in vivo virulence, mice were anesthetized by subcutaneous injection of Zoletil (50 mg/kg; Virbac, Carros, France) and subsequently infected intranasally (i.n.) with 2 × 10^3^ CFU of either the *M. bovis* empty vector control or the VapC40-overexpressing strain. For vaccine efficacy assessment, mice were subcutaneously (s.c.) immunized with 1 × 10^5^ CFU of either the BCG vaccine or the *vapC40* knockout strain. Four weeks post-immunization, the mice were challenged intranasally with 2 × 10^3^ CFU of wild-type *M. bovis*. Body weight was recorded weekly throughout the experimental period.

### 4.8. Bacterial Enumeration and Histopathological Analysis

At 28 days post-infection (dpi), mice were humanely euthanized, and the lungs and spleens were aseptically collected. The excised tissues were homogenized using ceramic beads (Shanghai Jingxin Industrial Development Co., Ltd., Shanghai, China) and serially diluted in sterile PBS containing 0.05% Tween-80. To determine the bacterial load, the tissue homogenates were plated in triplicate on Middlebrook 7H10 agar plates. After incubation at 37 °C for 3 weeks, colony-forming units (CFU) were enumerated.

For histopathological analysis, the left lung lobes were excised and fixed in 10% neutral buffered formalin. After paraffin embedding, tissue sections were cut at a thickness of 4–5 μm. Sections were stained with hematoxylin and eosin (H&E) to assess pathological changes and with Ziehl–Neelsen (ZN) staining to visualize acid-fast mycobacteria. Lung tissue sections were evaluated for inflammatory changes by two independent pathologists in a blinded manner. Inflammatory areas were defined based on the following criteria: (1) interstitial thickening with increased cellularity, (2) alveolar inflammatory cell infiltration including neutrophils, macrophages, and lymphocytes, (3) disruption of normal alveolar architecture, and (4) presence of inflammatory exudates. Stained slides were scanned using a Leica CS2 scanner (Leica Biosystems, Nussloch, Germany), and images were analyzed using Slide Viewer (v2.5.0; 3DHISTECH Ltd., Budapest, Hungary), ImageScope (v12.3; Leica Biosystems), and ImageJ software (v1.8.0; NIH, Bethesda, MD, USA).

### 4.9. Ifn-Γ Quantification by ELISA

At 28 days post-immunization, blood samples were collected from mice to obtain serum. In parallel, culture supernatants from antigen-stimulated splenocytes were collected. The concentrations of IFN-γ in both serum and supernatants were measured using a commercial Mouse IFN-γ ELISA Kit (Cat# MM-45169M1; Enma Industrial Co., Ltd., Yancheng, China) according to the manufacturer’s instructions. Briefly, samples and standards were added to the pre-coated microplate and incubated with a horseradish peroxidase (HRP)-conjugated detection antibody. After washing, chromogenic substrate was added, and the plate was incubated in the dark. The enzymatic reaction was then stopped, and the optical density (OD) at 450 nm was measured using a microplate reader. Cytokine concentrations were calculated based on a standard curve generated using recombinant mouse IFN-γ standards.

### 4.10. Flow Cytometry and Intracellular Cytokine Staining

To evaluate antigen-specific T cell responses, single-cell suspensions were prepared from the spleens of immunized mice. Approximately 1 × 10^6^ splenocytes were plated per well and stimulated with antigen in the presence of a protein transport inhibitor for 8 h. To prevent nonspecific binding, cells were incubated with an anti-mouse CD16/CD32 (Cat# E-AB-F0997A; Elabscience, Wuhan, China) Fc receptor-blocking antibody on ice for 10 min. Surface staining was then performed by incubating cells with V450-conjugated anti-CD3 (Cat# E-AB-F1013Q; Elabscience, Wuhan, China) and FITC-conjugated anti-CD4 (Cat# E-AB-F1097C; Elabscience, Wuhan, China) antibodies for 30 min at 4 °C in the dark. After washing with cell staining buffer, cells were fixed with fixation buffer for 30–60 min at room temperature, followed by permeabilization with a 1× permeabilization buffer. Intracellular staining was performed using an APC-conjugated anti-IFN-γ antibody (Cat# E-AB-F1101E; Elabscience, Wuhan, China) for 30 min at room temperature in the dark. Cells were then washed, resuspended in buffer, and analyzed using a flow cytometer. Data were processed using FlowJo software.v10.08.1.

### 4.11. Statistical Analysis

Statistical analyses were performed using GraphPad Prism 8.0 software (GraphPad Software, San Diego, CA, USA). Data are presented as the mean ± standard deviation (SD) from at least three independent biological replicates. Differences between the two groups were analyzed using an unpaired two-tailed Student’s *t*-test. For comparisons involving three or more groups, one-way ANOVA was performed, followed by Tukey’s post hoc test for multiple comparisons. *p* < 0.05 was considered statistically significant.

## Data Availability

The original contributions presented in this study are included in the article. Further inquiries can be directed to the corresponding authors.
